# Comparing the differences of prokaryotic microbial community between pit walls and bottom from Chinese liquor revealed by 16S rRNA gene sequencing

**DOI:** 10.1515/biol-2022-0571

**Published:** 2023-02-23

**Authors:** Shu Fang, Chuanxiang Wang, Juan Yan

**Affiliations:** School of Biological and Environmental Engineering, Chaohu University, Hefei 230000, China; Quality and Technology Department, Anhui Yun Distillery Group Co., Ltd, Ma’anshan 243000, China

**Keywords:** strong-flavor liquor, high-throughput sequencing, pit mud, prokaryotic microbial community, gene function prediction

## Abstract

This study aims to explore the prokaryotic microbial community structures and diversity in pit mud from different depths, and provide a theoretical basis for the liquor production and further study of pit mud. The fermented pit muds of strong-flavor liquor from Yun distillery were taken as samples. The high-throughput sequencing approach, followed by bioinformatics analyses, was used to compare the differences in the prokaryotic microbial community between pit walls and bottom represented by samples. A total of 31 bacteria phyla and 2 archaea phyla were detected. The dominant phyla in YJ-S, YJ-Z, and YJ-X (sample name) were *Proteobacteria* and *Firmicutes*, while the dominant genera in them were *Acinetobacter*, *Aminobacterium*, and *Lactobacillus*. YJ-Z and YJ-X were the closest in species diversity. In species richness analysis, YJ-X was the highest, followed by YJ-Z, and YJ-S was the lowest; in species uniformity analysis, YJ-S was the highest, followed by YJ-Z, and YJ-X was the lowest. The function predicted by 16S rRNA genome showed that prokaryotic microbial function in pit mud was mainly concentrated in “Carbohydrate transport and metabolism” and “Amino acid transport and metabolism.” Significant differences in prokaryotic microbial community and gene function prediction between pit walls and bottom were found in YJ-S, YJ-Z, and YJ-X (*p* < 0.05).

## 1 Introduction

China is a country with a long history of traditional liquor (baijiu) production. Baijiu is one of the world’s six major distilled liquors [1]. Liquor is mainly produced in the way of solid-state fermentation of certain crops, such as sorghum, corn, wheat, rice, and glutinous rice [2]. During the fermentation process of liquor, liquor has gradually developed with 12 kinds of different styles in the land of China, due to the differences in brewing raw materials, brewing technology, ecological environment, saccharification, distillation, and other factors in different regions of China, such as sauce-flavored, strong-flavored, light-flavored, rice-flavored, and medicinal flavors [3,4].

Among these liquors, strong-flavor liquor, which is produced by distilling fermented grains (mainly sorghum), has the strong fragrance, sweetness, and lasting aftertaste [1,2]. Strong-flavor liquor is the most popular type of liquor in China, accounting for more than half of the liquor consumption market [5,6]. During the production process of solid-state fermentation of traditional Chinese liquor, a bucket-shaped pit must be excavated underground in advance. The inner wall and bottom of the pit are coated with special fermented clay, which is called pit mud [7]. Pit mud consists of cooked sticky loess, humus soil, cooked bean cake powder, bran koji powder, yellow water, caproic acid bacteria solution, fragrant mud, yeast solution, ammonium phosphate, urea, and wine tail. The above materials are mixed and stirred in a certain proportion, then it is formed after a complex fermentation process in mother-pit. Pit mud is essential for the fermentation and production of strong-flavor liquor, due to the presence of many microorganisms, such as bacteria, archaea, and fungi [6,7]. These microorganisms, which can produce aromatic compounds for the fermentation of strong-flavor liquor, rely strongly on the suitable microecological environment provided by the pit mud [8]. Many studies have shown that the flavor of liquor is closely related to the microorganisms used for fermenting liquor raw materials in the pit mud [9]. The microbial community structure of pit mud is quite complex and closely related to the age and geographical location of the pit [4]. Different microorganisms ferment the raw materials and produce various enzymes and aromatic compounds in the liquor, forming various types of liquors with distinctive flavors [9,10].

In order to increase the output of liquor, liquor-production enterprises generally build larger pits. The pit provides a closed environment for microbial fermentation in pit mud and Daqu. The temperature in the environment is 25–32°C, the humidity is 40–45%, and the pH is 3.0–5.0 [5]. Due to the differences in the microecological environment at different locations of the pit, such as pit walls and bottom, there are differences in the microbial community abundance at different locations in the pit, which leads to differences in the quality of the fermented liquor, and affects the quality of liquor production; such studies have rarely been reported [4].

In this study, Anhui Yun distillery Group Co., Ltd, located in Ma’anshan city, east of Anhui province, China, established some pits with 2.85 m in length, 2.65 m in width, and 2.2 m in depth for the production of strong-flavor liquor in 2003, the pit muds of the pits were cultivated in the same microorganism enriched mother-pit. Since the quality of liquor produced in these pits was not exactly the same, the pit producing the best quality liquor was selected as our research subject by consulting the distillery technician. High-throughput sequencing technologies have developed rapidly and emerged as an effective tool, both for the microbial diversity analysis of complex samples and studying metabolic pathways in recent years [11–14]. The technique of high-throughput sequencing has also been recently used for microbial community structure analysis of Chinese liquor [2,4–6,10,15]. In prokaryotic cells, the 16S rRNA genes are composed of variable regions and conserved regions, the former of which can be sequenced to identify between microbes for bacterial phylogenetic and taxonomic identification [11]. In this study, the variables V3 and V4 regions of the 16S rRNA genes were used to determine the prokaryotic microbial diversity in pit walls and bottom. Our study provides a theoretical basis for the daily maintenance of pit mud, the specifications of new pits construction, and the quality of liquor production.

## 2 Materials and methods

### 2.1 Sample collection

**Figure 1:**
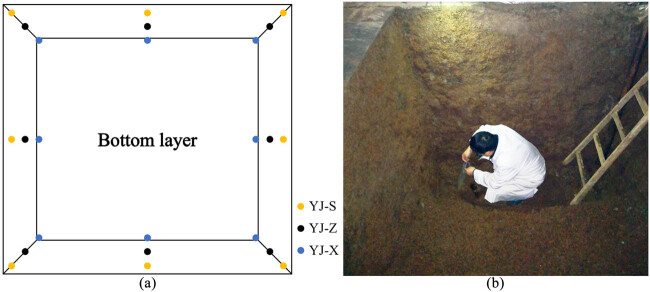
Sampling points of mud in the pit: (a) sampling diagram and (b) sampling in the pit.

After the selected pit was opened and the distiller’s grains were taken out, the pit muds in 24 positions (1 g/each sample position) were taken vertically and equidistant from the fermentation pit in upper (0.2 m in depth), middle (1.2 m in depth), and bottom layer (2.2 m in depth), respectively (Figure 1). The pit muds in the same layer (eight positions/each sample layer) were transferred into a sterile bag, sealed, named as YJ-S, YJ-Z, YJ-X, and then stored at −80°C until further analysis.

### 2.2 DNA extraction

Pit mud samples needed to be transformed into pellets through pretreatment, then the resulted pellets from the three samples were performed using centrifuge following the method described by Ding et al. [16]. The pellets were subjected to DNA extraction using the commercial Mag-Bind Soil DNA Kit (Omega Bio-tek Corporation, USA) according to the manufacturer’s instruction. The concentration and purity of extracted DNAs were measured using 0.8% (w/v) agarose gel electrophoresis and a micro-spectrophotometer, respectively. The resulted DNA was diluted to a DNA concentration of 1 ng/μL and subsequently used as a template for PCR to amplify 16S rRNA genes.

### 2.3 PCR amplification

The V3 and V4 regions of 16S rRNA genes were amplified using the universal forward primer 341F(5′-CCCTACACGACGCTCTTCCGATCTG(barcode)CCTACGGGNGGCWGCAG-3′) and the reverse primer 805R (5′-GACTGGAGTTCCTTGGCACCCGAGAATTCCAGACTACHVGGGTATCTAATCC-3′) with a set of six-nucleotide barcodes, the primers were modified and synthesized by Sangon Biotech Company (Shanghai, China) [17,18]. The detailed PCR procedures were modified according to a method described by Tang et al. [19]. The PCR amplification conditions were as follows: 94°C for 3 min; 94°C for 30 s, 45°C for 20 s, 65°C for 30 s, five cycles; 94°C for 20 s, 55°C for 20 s, 72°C for 30 s, 20 cycles and 72°C for 5 min. The amplified PCR products were detected by 2% agarose gel electrophoresis and purified using the commercial QIAquick Gel Extraction Kit (QIAGEN Corporation, Germany). After gel purification, the PCR product was quantified using the commercial Qubit ssDNA Assay Kit (Life Technologies, USA). Finally, the purified 16S rRNA gene amplicons were to construct DNA library using TruSeq DNA PCR-Free Sample Preparation Kit (Illumina Inc, USA) and pair-end sequenced on an Illumina HiSeq2500 PE250 platform at Sangon Biotech Company (Shanghai, China).

### 2.4 Genome data processing

The original image data file obtained by high-throughput sequencing was transformed into the original sequence by CASAVA base recognition analysis, which was called raw reads. Cutadapt software (V1.2.1) [20] was used to remove the primer splice sequence of raw reads, pear software (V0.9.6) [21] was used to splice sequences, Prinseq software (V0.20.4) [22] was used to filter sequences, Usearch software (V5.2.236) [23] was used to remove chimeras and nonspecific amplification sequences, and finally, the qualified sequences were obtained.

### 2.5 Bioinformatics analysis of the 16S rRNA genome sequences

Usearch software contains a suite of software tools that were used to cluster all effective tags to the operational taxonomic units (OTU) based on 97% identity of the sequences. The taxonomy of each OTU representative sequence was assigned using the Ribosomal Database Project (http://rdp.cme.msu.edu/misc/resources.jsp) classifier with a minimum bootstrap threshold of 80%. Subsequently, taxonomic information at the six-level classification level domain, phylum, class, order, family, and genus was obtained [24]. Based on the results of the OTU, a Venn diagram was generated using R software (V3.2) [25] to show the number of OTU of microorganisms that were shared in the three samples and the variance among them. The Bray–Curtis algorithm was used to construct a sample clustering tree and species abundance histogram based on the abundance of different sample species. Mothur software (V1.2.7) [26] was used to calculate the five kinds of alpha diversity index, such as Shannon, ACE, Chao1, Coverage, and Simpson; R software was used to draw dilution curve and rank abundance curve. Heat maps were drawn based on unifrac distance matrix between different samples; PICRUSt software (V1.1.4) [27] was used to predict the metagenomic function of the three samples, and abundance heat maps based on the COG function and the KEGG metabolic pathway were both drawn. STAMP software (V2.1.3) [28] was used to compare with the differences of species abundance and gene functional abundance among the three samples, the 25 ranks with the lowest *p* value were listed in the figures, when *p* was less than 0.05, the species classification was marked in red. In statistical analysis, significance was determined at 95% confidence interval (*p* = 0.05).

### 2.6 Nucleic acid sequence accession number

The 16S rRNA genome sequences obtained by high-throughput sequencing in this study had been submitted to NCBI database under the accession number: SRR15900602, SRR15900603, and SRR15900777.

## 3 Results

### 3.1 OTU clustering of microorganisms in pit mud

**Figure 2:**
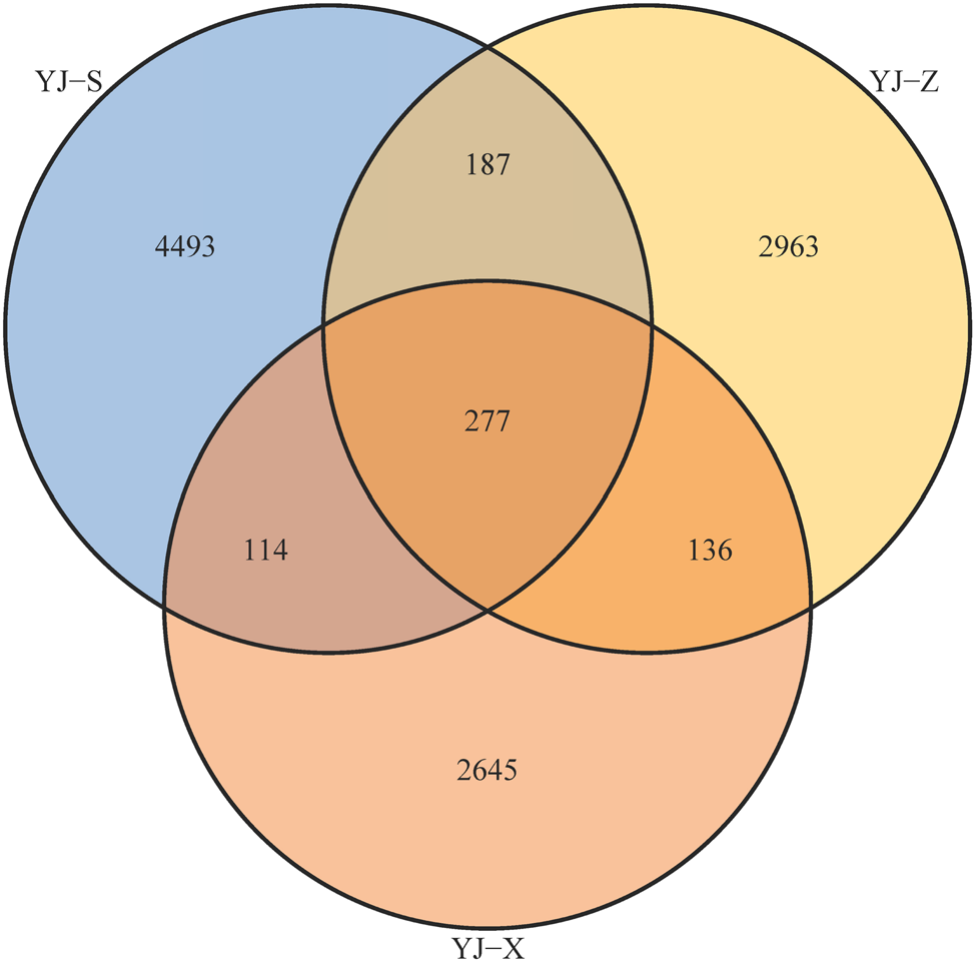
Venn diagram of number of OTUs of microorganism in pit mud.

After high-throughput sequencing of PCR amplification products, a total of 208,366 raw reads were obtained from the three samples, and 175,150 qualified reads were obtained after processing (Table 1). These 16S rRNA gene sequences were clustered into 11,806 OTUs for species analysis with 97% similarity. Among them, YJ-S had 58,282 qualified reads and a cluster of 5,071 OTUs, YJ-Z had 49,669 qualified reads and a cluster of 3,563 OTUs, YJ-X had 67,199 qualified reads and a cluster of 3,172 OTUs (Table 1). The Venn diagram can be used to count the number of shared and unique OTUs in samples. There were 464 OTUs in both YJ-S and YJ-Z, accounting for 3.93% of the total OTUs; 391 OTUs in both YJ-S and YJ-X, accounting for 3.31% of the total OTUs; and 413 OTUs in both YJ-Z and YJ-X, accounting for 3.50% of the total OTUs. There were 277 OTUs in YJ-S, YJ-Z, and YJ-X, accounting for 2.35% of the total OTUs (Figure 2). To sum up, YJ-S and YJ-Z had the highest similarity in OTU number composition, followed by YJ-Z and YJ-X, finally YJ-S and YJ-X.

**Table 1:** Sample sequencing information and alpha diversity of microorganism in pit mud

Sample	Raw reads	Qualified reads	OTU	Alpha diversity index				
				Shannon	ACE	Chao1	Coverage	Simpson
YJ-S	73,608	58,282	5,071	3.65	90410.65	34646.12	0.93	0.17
YJ-Z	59,100	49,669	3,563	3.66	33985.33	23344.33	0.94	0.12
YJ-X	75,658	67,199	3,172	4.38	60690.04	22079.02	0.97	0.08

### 3.2 Taxonomic annotation of microorganisms in pit mud

**Figure 3:**
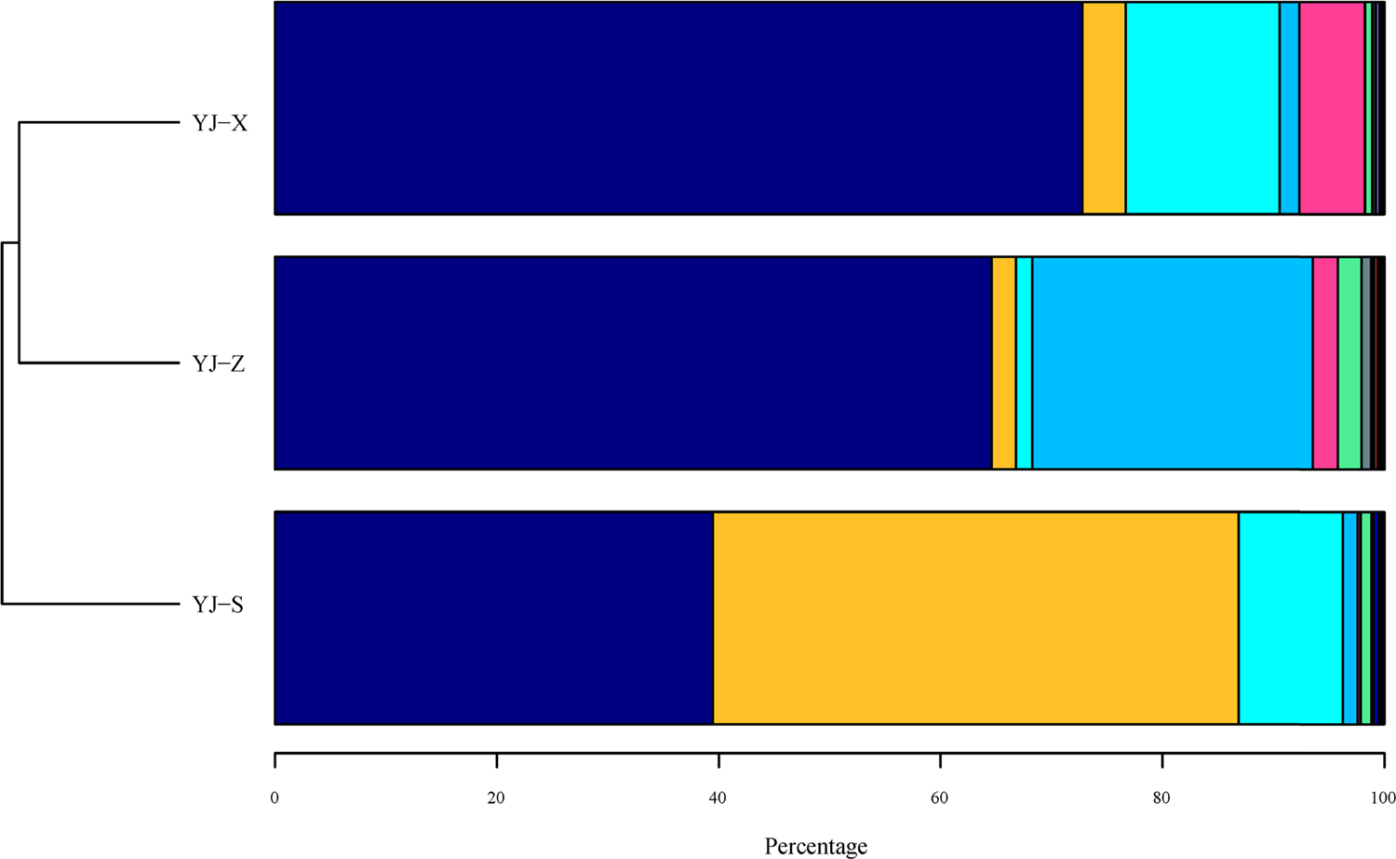
Phylum level distribution of microorganism population present in pit mud. Note: (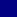) *Firmicutes*; (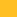) *Proteobacteria*; (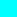) *Bacteroidetes*; (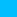) *Synergistetes*; (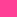) *Actinobacteria*; (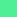) *Euryarchaeota*; (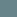) unclassified; (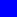) *Chloroflexi*; (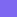) *Verrucomicrobia*; (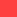) *Acidobacteria*; (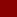) *Tenericutes*; (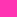) *Cloacimonetes*; (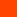) *Planctomycetes*; (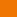) *Armatimonadetes*; (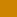) *Spirochaetes*; (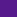) *Gemmatimonadetes*; (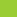) *Candidatus Saccharibacteria*; (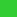) *Deinococcus-Thermus*; (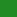) *Atribacteria*; (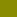) *Chlamydiae*; (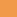) *Parcubacteria*; (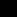) *Nitrospirae*; (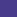) *Ignavibacteriae*; (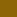) *Lentisphaerae*; (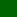) candidate division WPS-1; (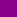) *Aminicenantes*; (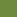) BRC1; (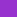) candidate division WPS-2; (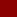) *Latescibacteria*; (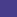) *Thermotogae*; (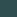) *Elusimicrobia*; (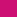) *Microgenomates*; (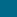) *Hydrogenedentes*; (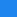) *Crenarchaeota*.

**Figure 4:**
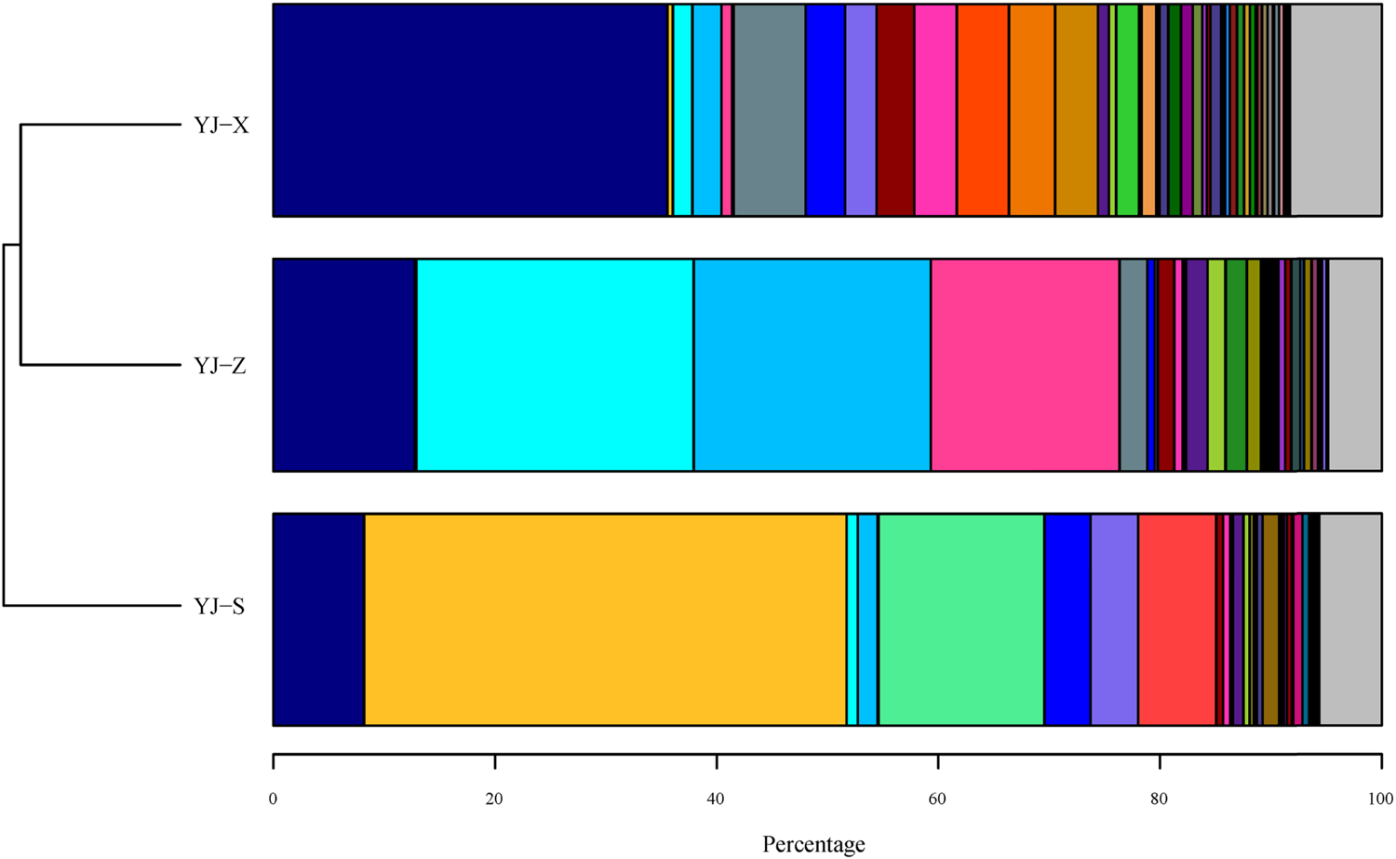
Genus level distribution of microorganism population present in pit mud. Note: (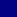) unclassified; (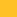) *Acinetobacter*; (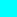) *Aminobacterium*; (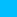) *Clostridium* IV; (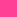) *Caloramator*; (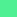) *Lysinibacillus*; (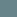) *Lactobacillus*; (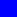) *Proteiniphilum*; (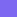) *Petrimonas*; (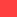) *Caryophanon*; (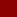) *Syntrophomonas*; (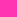) *Clostridium sensu stricto*; (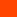) *Bifidobacterium*; (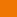) *Bacteroides*; (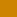) *Faecalibacterium*; (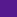) *Sedimentibacter*; (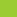) *Tissierella*; (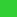) *Blautia*; (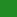) *Olsenella*; (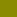) *Garciella*; (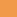) *Alistipes*; (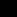) *Syntrophaceticus*; (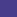) *Clostridium* III; (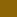) *Solibacillus*; (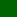) *Roseburia*; (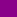) *Burkholderia*; (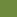) *Clostridium* XlVa; (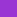) *Lutispora*; (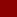) *Methanoculleus*; (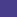) *Gemmiger*; (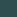) *Methanomassiliicoccus*; (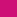) *Rummeliibacillus*; (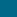) *Bacillus*; (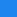) *Sporanaerobacter*; (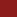) *Collinsella*; (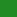) *Anaerostipes*; (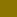) *Methanobacterium*; (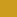) *Oscillibacter*; (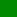) *Lachnospiracea*_*incertae*_*sedis*; (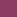) *Tepidimicrobium*; (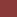) *Intestinimonas*; (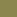) *Barnesiella*; (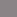) *Parabacteroides*; (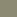) *Klebsiella*; (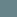) *Fusicatenibacter*; (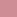) *Ralstonia*; (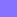) *Pelotomaculum*; (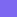) *Tepidanaerobacter*; (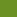) *Anaerovorax*; (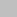) other.

Both bacteria and archaea were detected in YJ-S, YJ-Z, and YJ-X. The abundance of bacteria and that of archaea in YJ-S were 99 and 1%, respectively, in YJ-Z 98% and 2%, respectively, and in YJ-X 99% and 0.7% ,respectively. Among the three samples, bacterial abundance was absolutely dominant compared with archaea. At the phylum level, a total of 33 phyla including *Firmicutes*, *Proteobacteria*, *Bacteroidetes*, *Synergistetes*, and *Actinobacteria* were identified in the 16S rRNA gene sequences of the three samples, and the abundances were significantly different (Figure 3; Figures S1–S3 in Supplementary 1, *p* < 0.05). In addition, *Euryarchaeota* and *Crenarchaeota* were both identified in all three samples, belonging to the Archaea phylum, but only the abundance of *Euryarchaeota* was significantly different (Figure 3; Figures S1–S3 in Supplementary 1, *p <* 0.05). *Firmicutes* was the prominent phylum in YJ-Z and YJ-X with the most abundances, while it was *Proteobacteria* in YJ-S (*p <* 0.05). At the genus level, the distribution of microbial communities in YJ-S, YJ-Z, and YJ-X was also different, and the abundance of different genera was also different. Having abundance levels of more than 1%, the main microorganisms in YJ-S were *Acinetobacter* (43.53%), *Lysinibacillus* (14.96%), *Caryophanon* (7.05%), *Petrimonas* (4.29%), *Proteiniphilum* (4.13%), *Clostridium* IV (1.79%), *Solibacillus* (1.5%), and *Aminobacterium* (1.03%), and 8.18% of the microorganisms were unclassified genus (Table 2). The main microorganisms in YJ-Z were *Aminobacterium* (25.04%), *Clostridium* IV (21.39%), *Caloramator* (17.01%), *Lactobacillus* (2.49%), *Semimentibacter* (1.93%), *Olsenella* (1.91%), *Tissierella* (1.6%), *Syntrophimonas* (1.48%), *Garciella* (1.25%), *Syntrophaceticus* (1.09%), and unclassified genus (12.75%) (Table 2). The main microorganisms in YJ-X were *Lactobacillus* (6.49%), *Bifidobacterium* (4.71%), *Bacteroides* (4.16%), *Faecalibacterium* (3.88%), *Clostridium sensu stricto* (3.83%), *Proteiniphilum* (3.55%), *Syntrophomonas* (3.41%), *Petrimonas* (2.83%), *Clostridium* IV (2.63%), *Blautia* (2.06%), *Aminobacterium* (1.75%), *Alistipes* (1.32%), *Roseburia* (1.14%), *Burkholderia* (1.07%), and unclassified genus (35.54%) (Table 2). To sum up, the numbers of genera that have an abundance of 1% or higher in YJ-S, YJ-Z, and YJ-X were 9, 10, and 14, respectively, each making up 78.28, 75.19, and 42.83% of the total abundance in the respective sample (Figure 4, Table 2). The abundances of genera mentioned above were significantly different in YJ-S, YJ-Z, and YJ-X (*p* < 0.05), and the *p* value is shown in Supplementary 2.

**Table 2:** Abundance (%) of major genera (≥1%) from each pit mud sample

Genus	YJ-S (%)	Genus	YJ-Z (%)	Genus	YJ-X (%)
*Acinetobacter*	43.53	*Aminobacterium*	25.04	*Lactobacillus*	6.49
*Lysinibacillus*	14.96	*Clostridium* IV	21.39	*Bifidobacterium*	4.71
*Caryophanon*	7.05	*Caloramator*	17.01	*Bacteroides*	4.16
*Petrimonas*	4.29	*Lactobacillus*	2.49	*Faecalibacterium*	3.88
*Proteiniphilum*	4.13	*Sedimentibacter*	1.93	*Clostridium sensu stricto*	3.83
*Clostridium* IV	1.79	*Olsenella*	1.91	*Proteiniphilum*	3.55
*Solibacillus*	1.50	*Tissierella*	1.60	*Syntrophomonas*	3.41
*Aminobacterium*	1.03	*Syntrophomonas*	1.48	*Petrimonas*	2.83
		*Garciella*	1.25	*Clostridium* IV	2.63
		*Syntrophaceticus*	1.09	*Blautia*	2.06
				*Aminobacterium*	1.75
				*Alistipes*	1.32
				*Roseburia*	1.14
				*Burkholderia*	1.07

### 3.3 Microbial diversity in the pit mud

**Figure 5:**
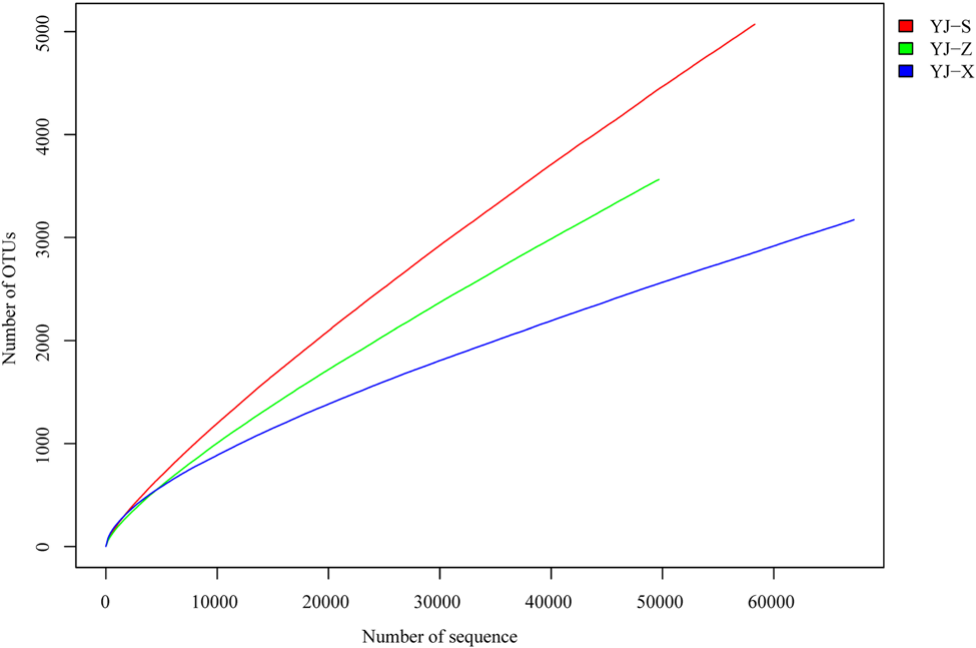
Dilution cure analysis of samples.

**Figure 6:**
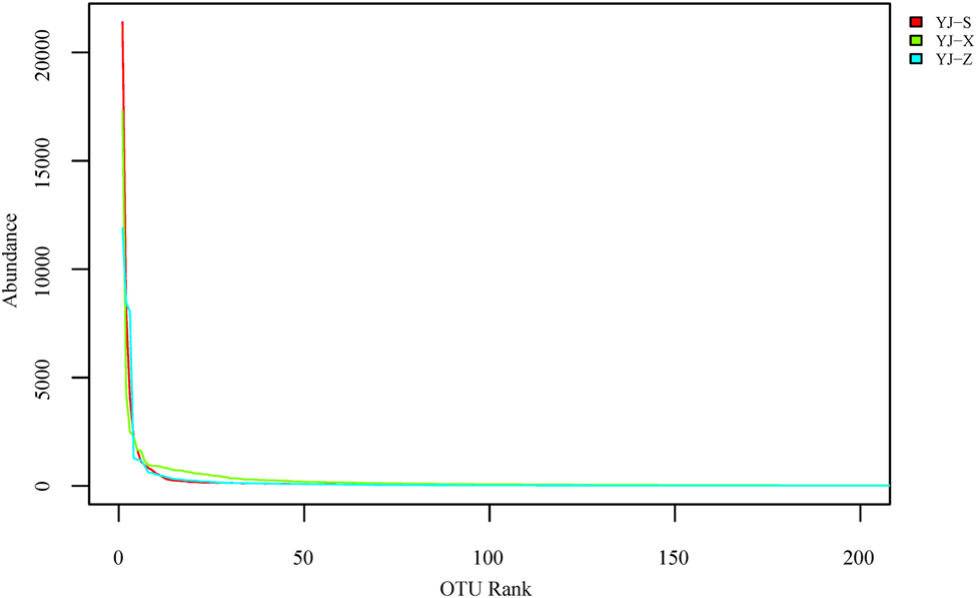
Rank abundance cure analysis of samples.

**Figure 7:**
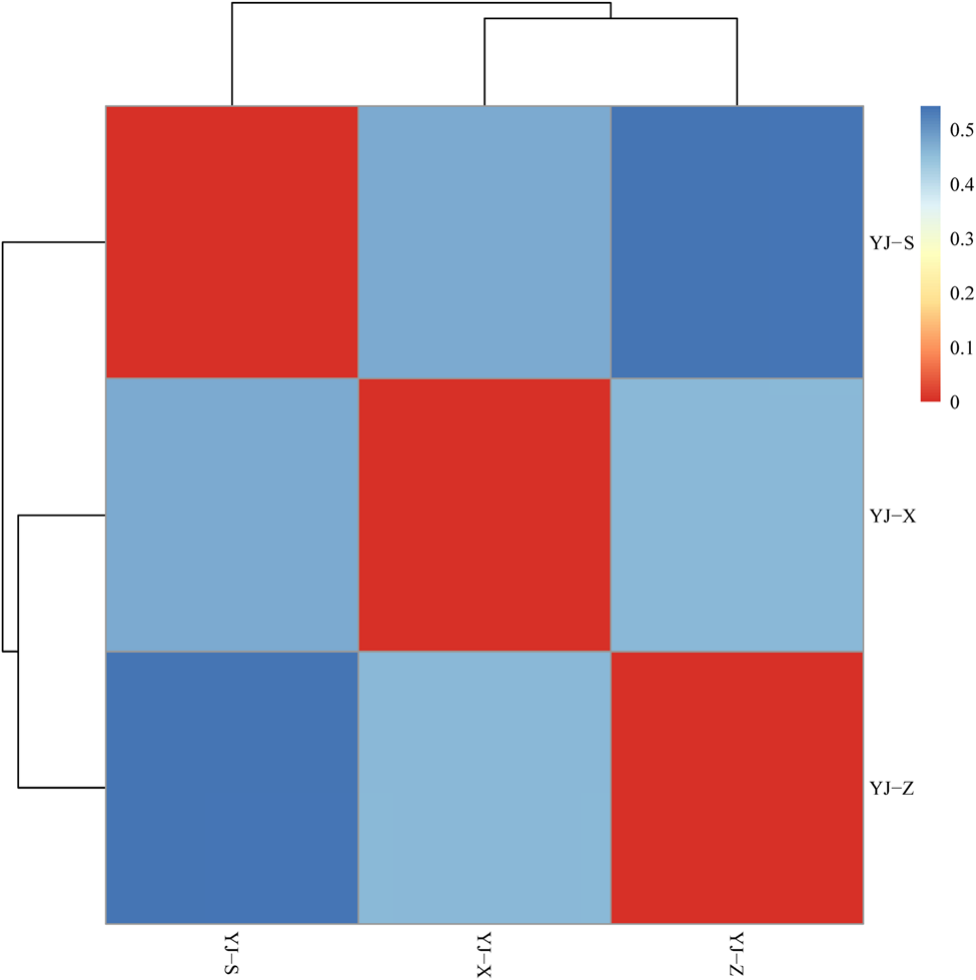
Distance heatmap of samples.

In the analysis of alpha diversity, diversity index, dilution curve, and rank abundance curve are important tools for the analysis of species diversity in different samples. The results from the Shannon index showed YJ-X (4.38) > YJ-Z (3.66) > YJ-S (3.65), and Simpson index showed YJ-X (0.08) < YJ-Z (0.12) < YJ-S (0.17) (Table 1). In microbial diversity between pit walls and bottom, the sample with the richest abundance was YJ-X, followed by YJ-Z and finally YJ-S (Figure 5). The rank abundance curve showed the species evenness: the highest being YJ-S, followed by YJ-Z and YJ-X (Figure 6). In the analysis of beta diversity, the weighted unifrac distance matrix between the two samples was used to draw a heat map for measuring the difference of the two samples. YJ-Z and YJ-X were the most similar in microbial diversity, while YJ-S and YJ-Z were the most different (Figure 7).

### 3.4 Gene functional diversity of microorganisms in pit mud

**Figure 8:**
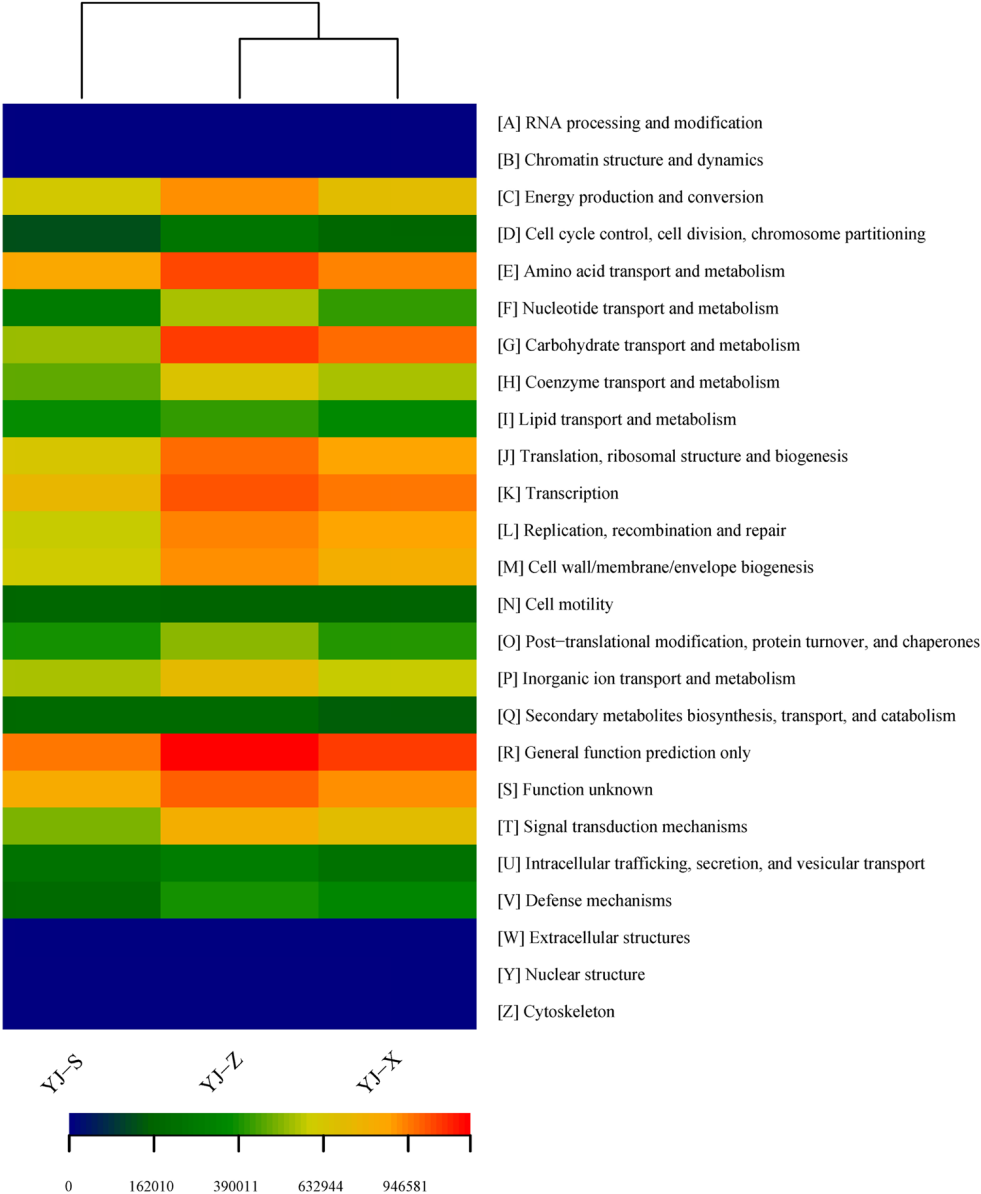
Heatmap of gene functional predictions of samples based on COG.

In COG analysis, the gene functional abundance ranked the top five in the three samples: “General function prediction only” followed by “Amino acid transport and metabolism,” “Function unknown,” “Transcription” and “Translation, ribosomal structure and biogenesis” in YJ-S; “General function prediction only” followed by “Carbohydrate transport and metabolism,” “Amino acid transport and metabolism,” “Transcription” and “function unknown” in YJ-Z; “General function prediction only” followed by “Carbohydrate transport and metabolism,” “Transcription,” “Amino acid transport and metabolism” and “Function unknown” in YJ-X (Figure 8, Table 3). And the abundances of gene functional were significantly different (Figures S4–S6 in Supplementary 1, *p* < 0.05).

**Table 3:** Abundance of top five COG classification of microorganism in pit mud

YJ-S	Abundance	YJ-Z	Abundance	YJ-X	Abundance
General function prediction only	1265413	General function prediction only	2064464	General function prediction only	1620397
Amino acid transport and metabolism	922062	Carbohydrate transport and metabolism	1615582	Carbohydrate transport and metabolism	1271587
Function unknown	881872	Amino acid transport and metabolism	1518171	Transcription	1194181
Transcription	790853	Transcription	1426763	Amino acid transport and metabolism	1158436
Translation, ribosomal structure and biogenesis	698184	Function unknown	1420056	Function unknown	1095976

**Figure 9:**
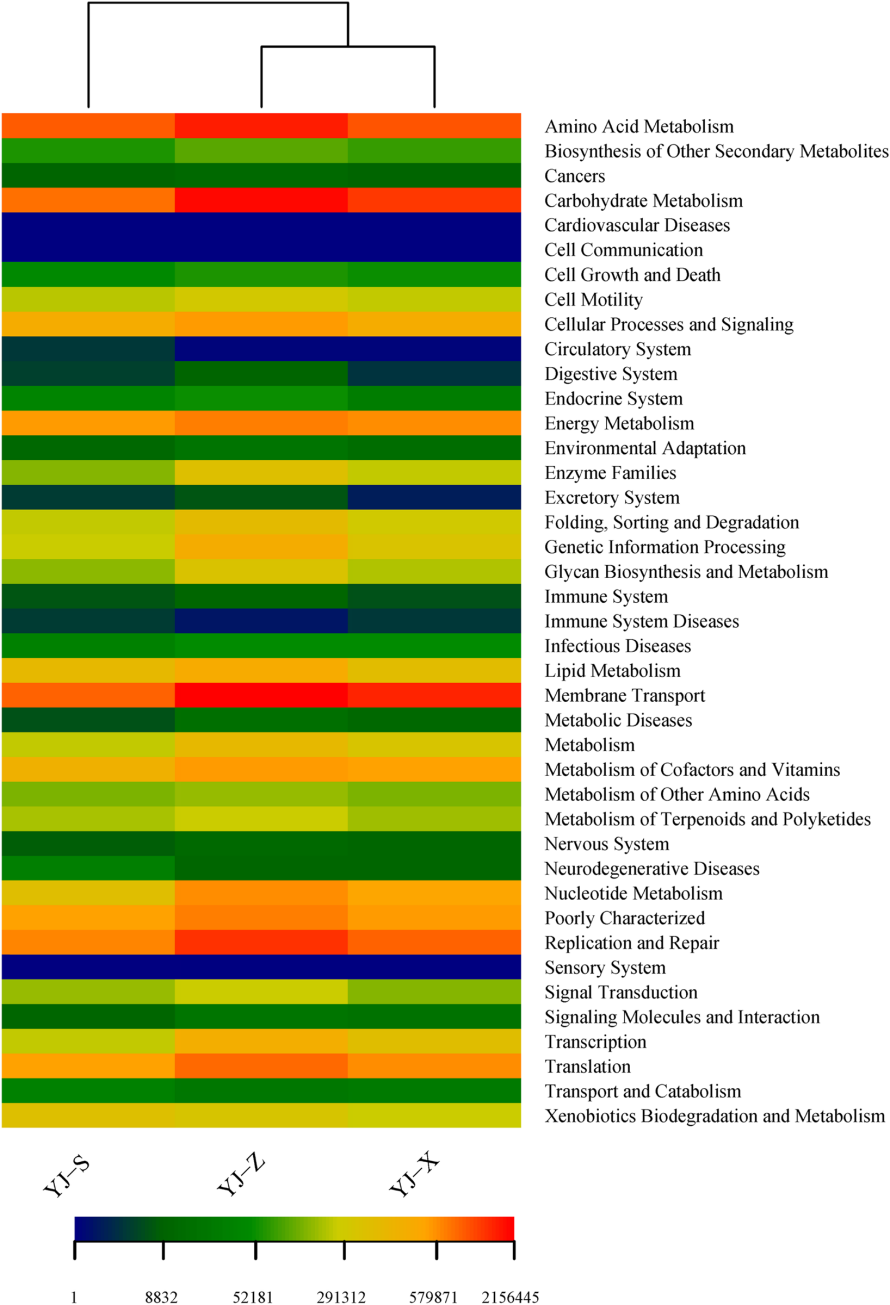
Heatmap of gene functional predictions of samples based on KEGG.

In the analysis of KEGG metabolic pathway, the gene functional abundance ranks in the top five: in YJ-S it was “Amino acid Metabolism” followed by “Membrane Transport,” “Carbohydrate Metabolism,” “Replication and Repair” and “Energy Metabolism.” In YJ-Z it was “Membrane Transport” followed by “Carbohydrate Metabolism,” “Amino Acid Metabolism,” “Replication and Repair” and “Translation.” In YJ-X it was “Membrane Transport” followed by “Carbohydrate Metabolism,” “Amino Acid Metabolism,” “Replication and Repair” and “Translation” (Figure 9, Table 4). And the abundances of gene function were significantly different (Figures S7–S9 in Supplementary 1, *p* < 0.05).

**Table 4:** Abundance of top five KEGG metabolic pathways of microorganism in pit mud

YJ-S	Abundance	YJ-Z	Abundance	YJ-X	Abundance
Amino acid metabolism	1245752	Membrane transport	2156445	Membrane transport	1765974
Membrane transport	1234878	Carbohydrate metabolism	1584091	Carbohydrate metabolism	2080906
Carbohydrate metabolism	1077914	Amino acid metabolism	1871697	Amino acid metabolism	1360333
Replication and repair	883471	Replication and repair	1677025	Replication and repair	1211644
Energy metabolism	654153	Translation	1142174	Translation	803222

## 4 Discussion

In recent years, microbial diversity in different types of fermented foods, such as fermented seafood, kimchi, traditional fermented mustard, traditional Myanmar fermented tea leaves, and traditional fermented vegetables, were all investigated using rRNA gene sequencing technology [13,14,29–31]. Based on the analysis of coverage index (≥0.93, Table 1) and dilution curve (Figure 5), all microorganisms in the three samples were covered relatively comprehensive, which could truly reflect the prokaryotic microbial community differences between pit walls and bottom. The results showed that YJ-Z and YJ-X were the most similar in terms of microbial diversity. However, due to the differences in vertical height and the microecological environment, the prokaryotic microbial community structure and abundance in YJ-Z and YJ-X were different. At the phylum level, the dominant phylum of YJ-Z and YJ-X was *Firmicutes*, while in YJ-S it was *Proteobacteria*. Previous studies also showed that *Firmicutes* and *Bacteroidetes* were the dominant phyla in pit muds [2,6,32–34]. *Firmicutes* can produce endophytic spores and resist extreme environments, degrade volatile fatty acids such as butyrate and its analogs, and is represented mainly by the *Clostridia* and *Bacilli* [10]. Although the relative abundance of *Firmicutes*, *Proteobacteria*, *Bacteroidetes*, and *Synergistetes* differed among YJ-S, YJ-Z, and YJ-X, they were still the key components of the microbiota in the pit mud of Yun liquor. And the similar results were also reported in the pit mud of Yibin liquor [2]. *Bacteroidetes* was the major contributor to the synthesis of terpenes [32]. In general, at increased depth within the pit mud, the relative abundance of *Firmicutes* and *Actinobacteria* shows an upward trend. At the genus level, the dominant genus of the three samples were also different. For example, *Aminobacterium* was the dominant genus in YJ-Z, with an abundance of 25.04%; while in YJ-S and YJ-X, *Aminobacterium*’s abundance was much lower, being 1.03% and 1.75%, respectively. *Aminobacterium* is a gram-negative, single arrangement, slightly curved rod, no spore formation, non-motile, and strictly anaerobic mesophilic bacteria. It can increase the content of ammonium nitrogen in pit mud by amino acid fermentation, which was first found in the sludge of the wastewater treatment tank of the dairy factory, and the model species of this bacterium was *Aminobacterium colombense* [35,36]. In this study, the abundance of *Aminobacterium* was the highest in YJ-Z, which may be related to the anaerobic environment in the middle of the pit and the fermentation temperature suitable for the growth of *Aminobacterium*. *Aminobacterium* and *Sedimentibacter* can ferment amino acids to generate ammonium nitrogen through metabolism, which could provide nitrogen sources for the growth of other microbes [10]. In YJ-X, the abundance of unclassified genus was as high as 35.54%, the dominant genus was *Lactobacillus*, and its abundance was only 6.49%; the abundance of *Lactobacillus* in YJ-Z was also as low as 2.49%; the abundance is the lowest in YJ-S, being only 0.03%. *Lactobacillus* is a gram-positive, rod-shaped, non-spore producing, facultative anaerobic, and fermenting with glucose to produce lactic acid. Lactic acid is further converted to ethyl acetate through enzymatic reaction, which increases the mellow smell of liquor. The repulsion relationship *Lactobacillus* has with other bacteria contributes to the production of caproic acid, which inhibits the growth of bacteriocin by pathogenic microorganisms and mold rot microorganisms, maintaining the quality of liquor fermentation [37,38]. *Lactobacillus* was accounted for the most in YJ-X, which was consistent with the reported viewpoints of Gao et al. [6]. *Lactobacillus* was critical for liquor brewing, also as a dominant genus among liquor pit muds of several flavor types [2]. *Acinetobacter* was the dominant genus in YJ-S, and the abundance was as high as 43.53%. *Acinetobacter* is a gram-negative, oxidase negative, and non-motile aerobic bacterium, which is widely distributed in soil and water, and even found in hot spring with an optimum growth temperature between 33 and 35°C [39,40]. *Acinetobacter* is the main microorganism for brewing maotai flavor and light flavor liquor by balancing the taste of alcohol and coordinating the effect of alcohol through the fermentation of glucose and other carbohydrates. In this study, *Acinetobacter* accounted for the main proportion in the upper part of the pit, indicating that YJ-S was partially similar to maotai flavor and light flavor in pit mud. The abundance of *Acinetobacter* in YJ-Z and YJ-X was very low, 0.14 and 0.51%, respectively. This may be caused by the fact that YJ-Z and YJ-X are further from the ground and have less contaction with oxygen, causing them to have different microecological environments than YJ-S. *Aminobacterium*, *Syntrophomonas*, and *Petrimonas* play a positive role in the maturation of pit mud, which indicate that the tested pit mud samples are in the mature state [10]. Based on the abundance of major genera in pit muds, the mature state of YJ-Z was more than YJ-S and YJ-X.

In this study, PICRUSt software was used to predict macrogenomic information from 16S rRNA gene of the three samples, and COG database and KEGG database were used to analyze microorganism gene COG classification and KEGG metabolic pathway in YJ-S, YJ-Z, and YJ-X, respectively. Since YJ-S, YJ-Z, and YJ-X were located in different microecological environments, their microorganism gene COG classification and KEGG metabolic pathway abundance were different from each other. In COG analysis, “General function prediction only” was in the first position in the three samples. The abundance of “Amino acid transport and metabolism” was decreased with the decrease of the vertical distance of the sample in the pit, which were in the second, third, and fourth positions in YJ-S, YJ-Z, and YJ-X, respectively. The function of “Amino acid transport and metabolism” may be related to the contact amount of oxygen. Further down the pit, the oxygen contact amount decreases, so the abundance of “Amino acid transport and metabolism” decreases. The abundance of “Function unknown” was the highest in YJ-S, and the abundance ranking was the same in YJ-Z and YJ-X. This may be due to the fact that YJ-S was closer to the ground and had more exposure to oxygen. “Carbohydrate transport and metabolism,” “Amino acid transport and metabolism,” and “Transcription” were the main genes function in YJ-S, YJ-Z, and YJ-X, which were consistent with the conclusion of Wang et al. [41] in studying the microbial community structure and gene function of high-temperature Daqu liquor in a factory in Xiangyang, Hubei Province. In KEGG analysis, “Amino Acid Metabolism” was in the first position in YJ-S, and in the third position in both YJ-Z and YJ-X. This may be due to YJ-S having a closer vertical distance to the ground, more contact with oxygen, more aerobic bacteria in pit mud, and more bacteria using amino acid fermentation. “Membrane Transport” and “Carbohydrate Transport and Metabolism” were in the second and third positions in YJ-Z and YJ-X, indicating that there may be a large number of cellulose degrading enzyme genes in the macrogenomes of the two samples, and a large number of cellulose degrading enzymes synthesized by bacteria were transported outside the cells to degrade cellulose in fermented grains. “Translation” was present at the fifth position in both YJ-Z and YJ-X, after the fifth position in YJ-S. It showed that the enzyme synthesis and metabolism of microorganism, and the characteristics that are beneficial to grain fermentation, in YJ-Z and YJ-X were stronger than those in YJ-S. “Carbohydrate Metabolism” and “Amino Acid Metabolism” were arranged at the second and third positions in YJ-Z and YJ-X, respectively, and the first and third positions in YJ-S, indicating that the gene functions of pit mud microorganism were mainly concentrated in carbohydrate metabolism and amino acid metabolism, which was consistent with the predicted function of “Amino acid transport and metabolism” and that of “Carbohydrate transport and metabolism.” The prediction was based on COG database in this study.

## 5 Conclusion

The study above showed that prokaryotic microbial community structures and diversity, and gene function prediction is significantly different in pit walls from those in the bottom. The dominant phyla in YJ-S, YJ-Z, and YJ-X were *Proteobacteria* and *Firmicutes*, and the dominant genera in them were *Acinetobacter*, *Aminobacterium*, and *Lactobacillus*. In species diversity analysis, YJ-Z and YJ-X were the closest; in species richness analysis, the order was YJ-X > YJ-Z > YJ-S; in species uniformity analysis, the order was YJ-S > YJ-Z > YJ-X. The prokaryotic microbial function in pit mud was mainly concentrated in “Carbohydrate transport and metabolism” and “Amino acid transport and metabolism.” These scientific data provide a theoretical basis for the future maintenance of pit mud, the specifications of building new pits, the input of fermentation material, and the quality of liquor production. In future, the study can be used as a groundwork for revealing the relationship among physicochemical properties, microbiome, and volatiles of pit mud.

## Supplementary Material

Supplementary Figure

Supplementary Table
