# Supplementary material for: Comparing the differences of prokaryotic microbial community between pit walls and bottom from Chinese liquor revealed by 16S rRNA gene sequencing
Source: Open Life Sci. 2023 Feb 23;18(1):20220571. doi: 10.1515/biol-2022-0571 (PMC9962418; doi:10.1515/biol-2022-0571)
Supplement: Supplementary Figure [file biol-2022-0571-sm.pdf]

Supplementary material

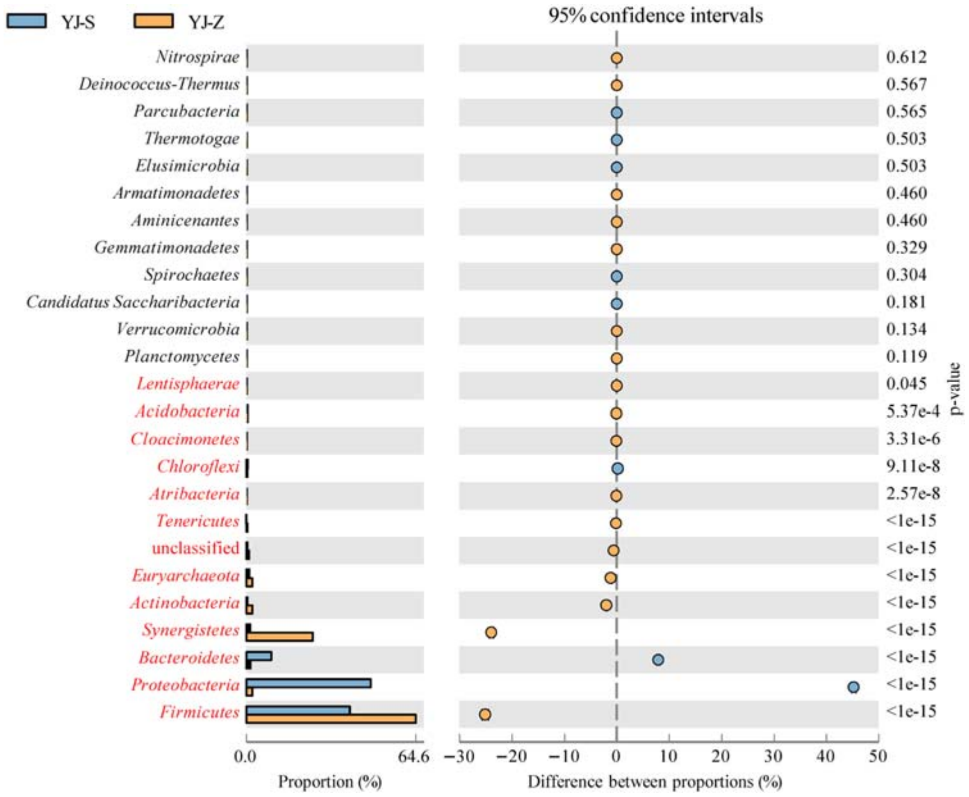

Figure S1: Phylum level difference of microorganism population between YJ-S and YJ-Z.

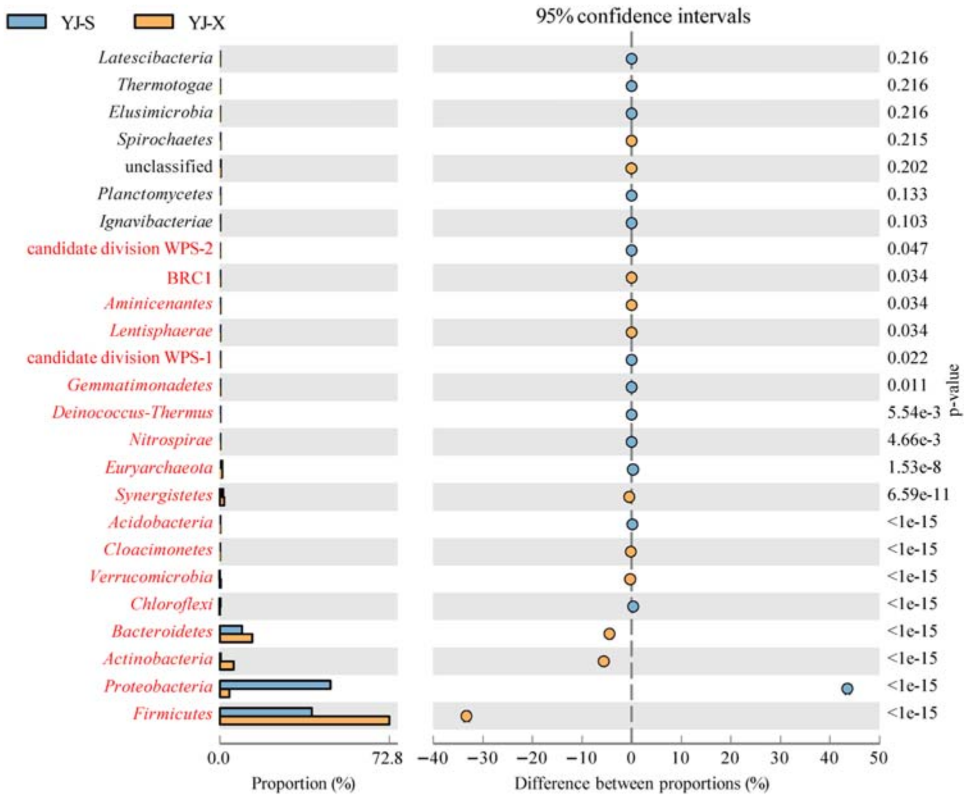

Figure S2: Phylum level difference of microorganism population between YJ-S and YJ-X.

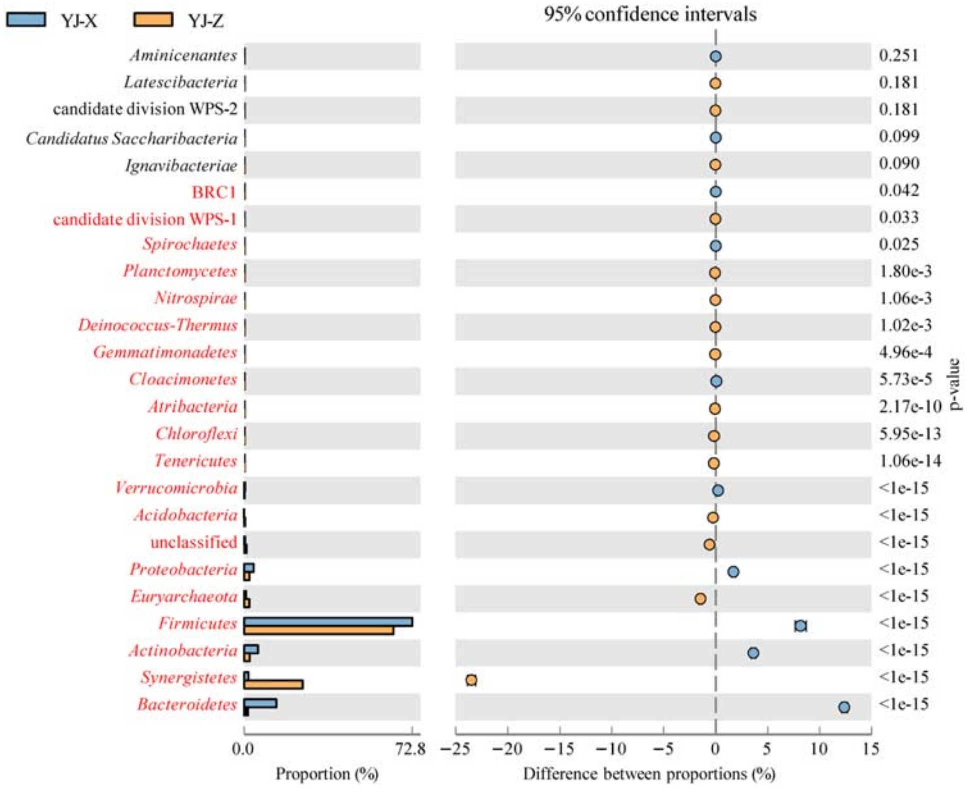

Figure S3: Phylum level difference of microorganism population between YJ-X and YJ-Z.

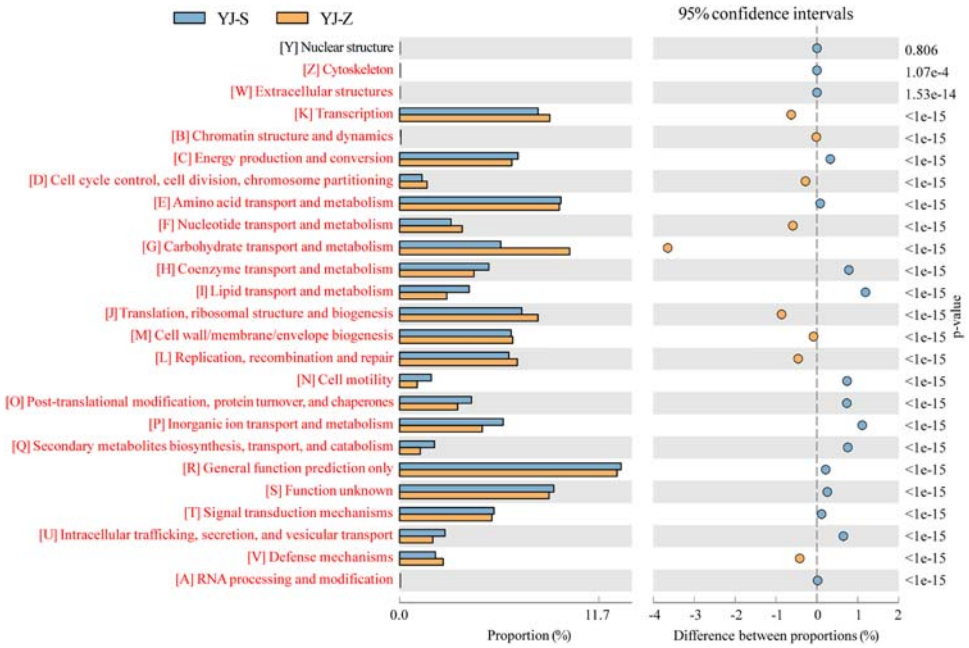

Figure S4: Difference of gene functional predictions between YJ-S and YJ-Z based on COG.

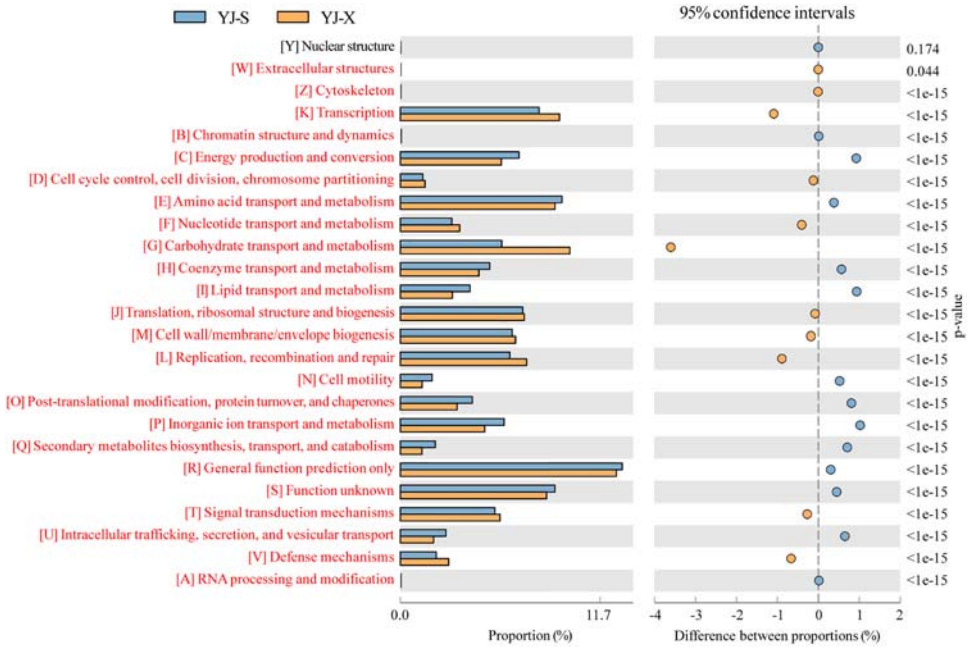

Figure S5: Difference of gene functional predictions between YJ-S and YJ-X based on COG.

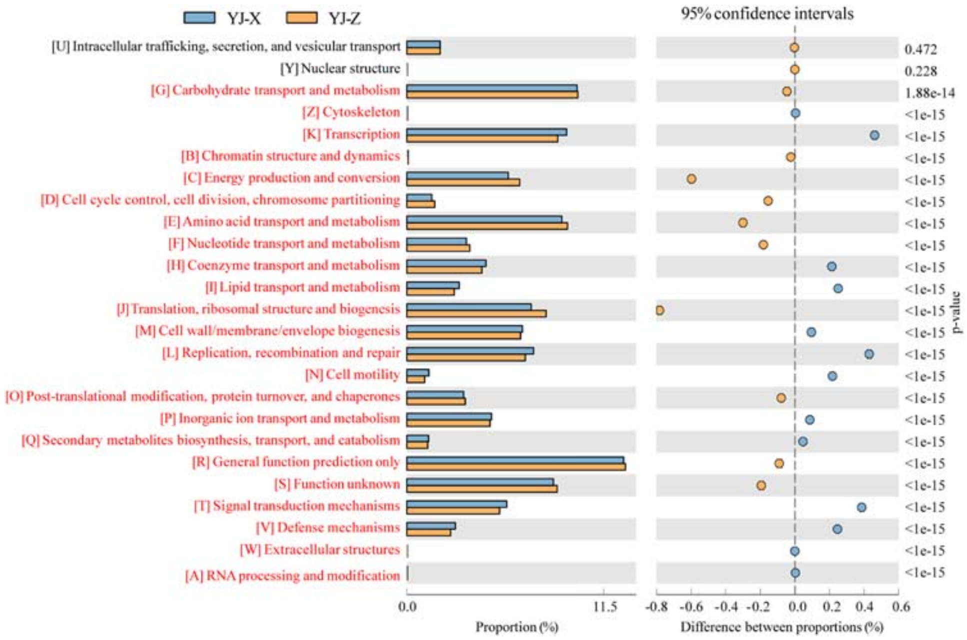

Figure S6: Difference of gene functional predictions between YJ-X and YJ-Z based on COG.

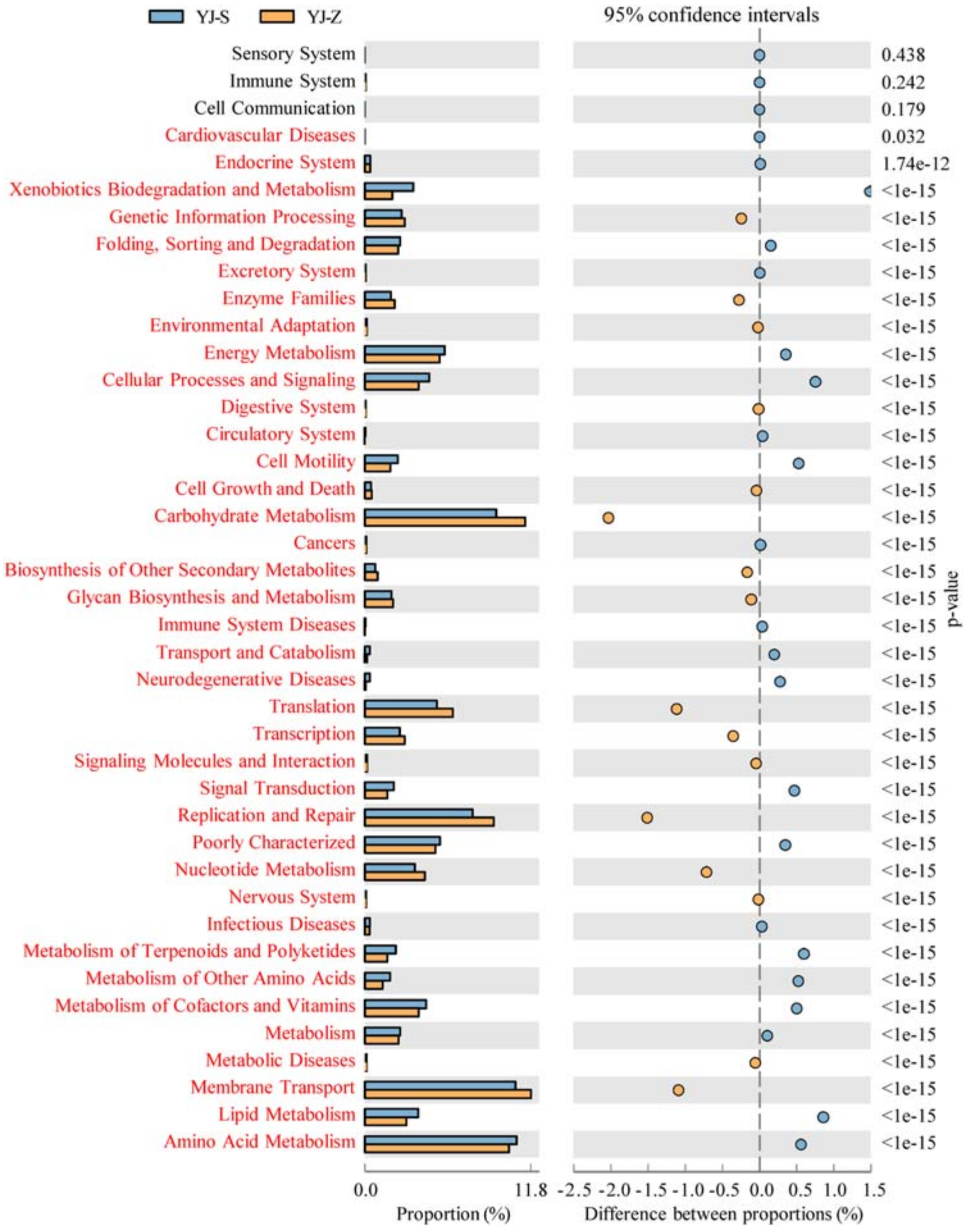

Figure S7: Difference of gene functional predictions between YJ-S and YJ-Z based on KEGG.

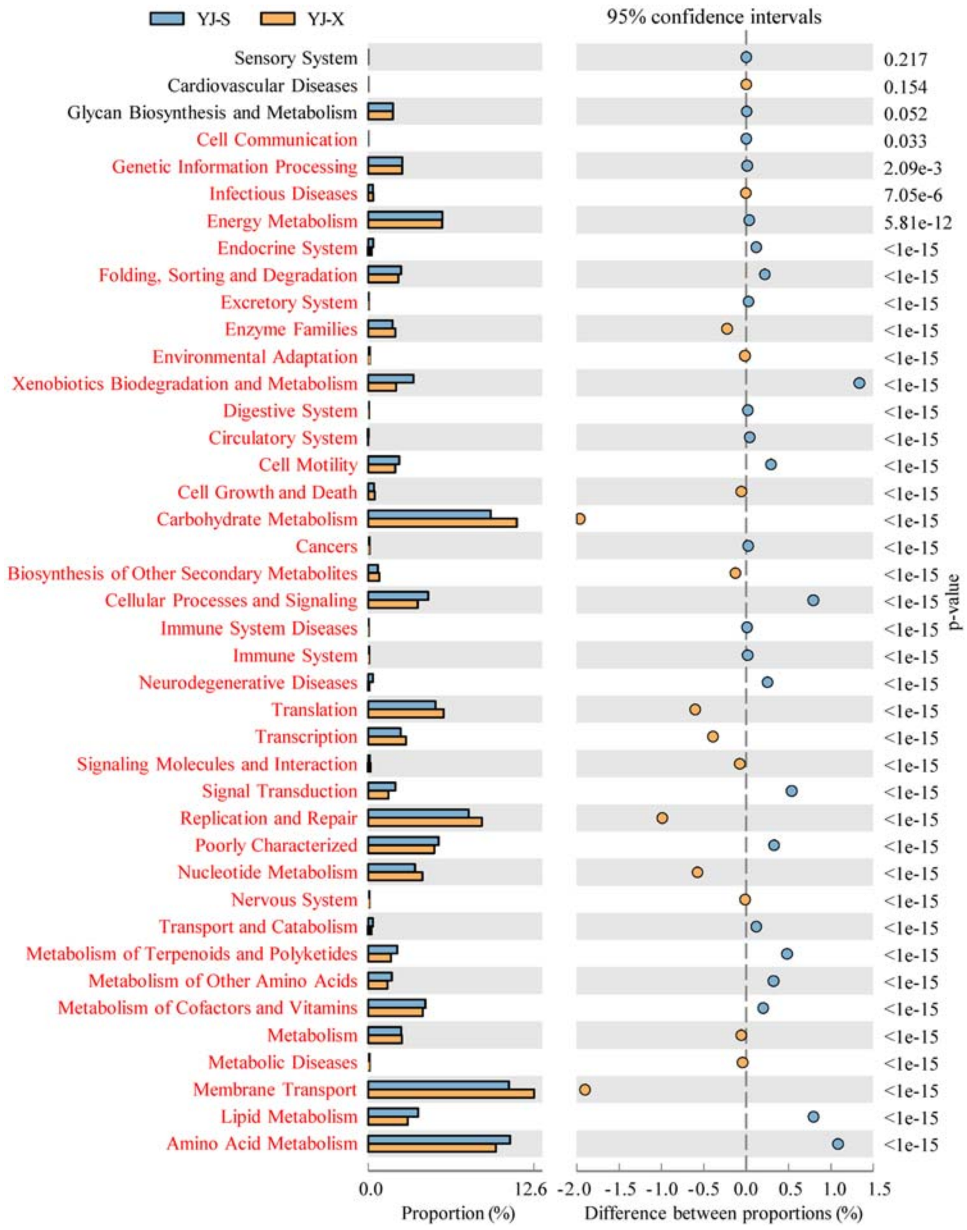

Figure S8: Difference of gene functional predictions between YJ-S and YJ-X based on KEGG.

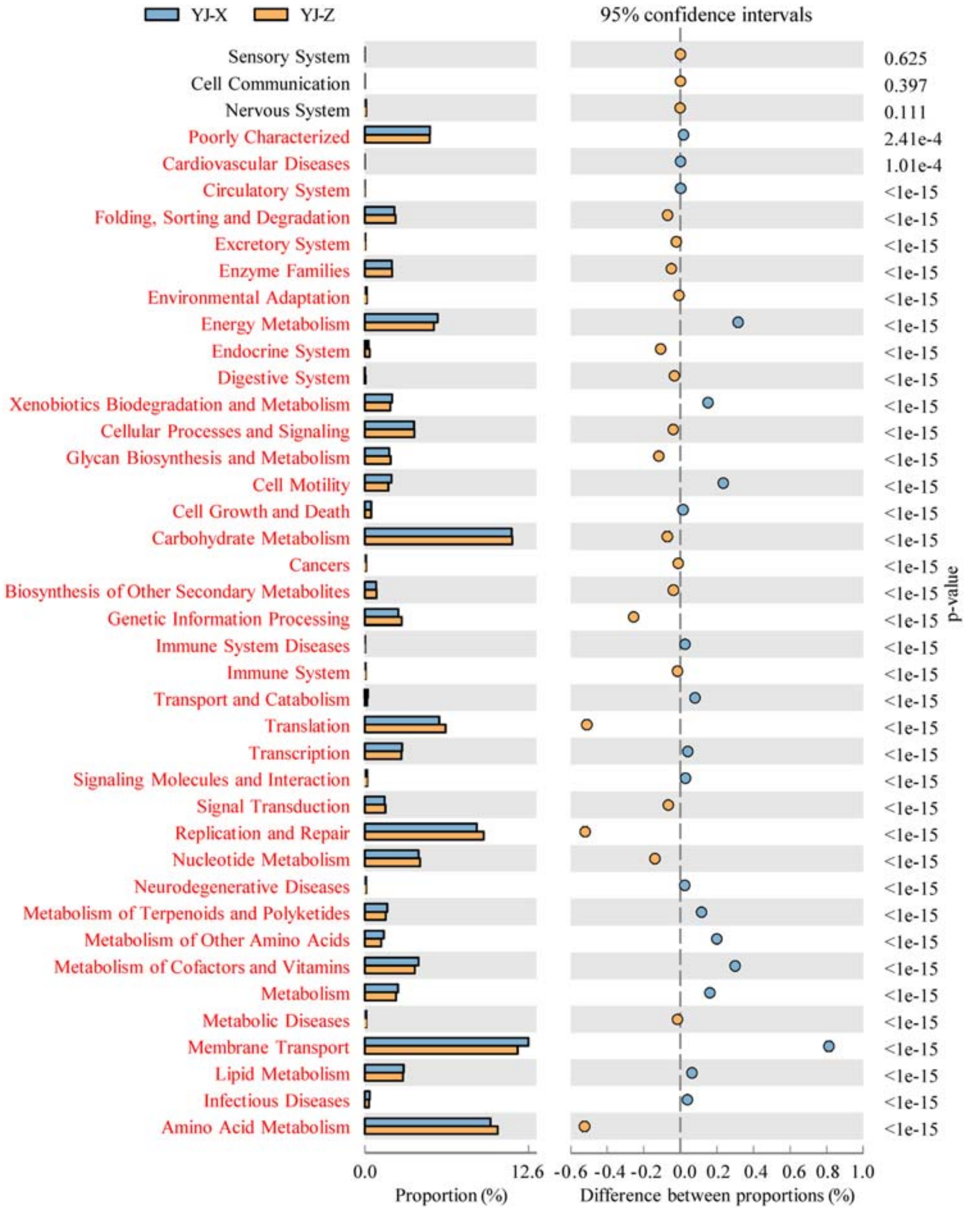

Figure S9: Difference of gene functional predictions between YJ-X and YJ-Z based on KEGG.
